# Dietary Tannic Acid Promotes Growth Performance and Resistance Against *Aeromonas hydrophila* Infection by Improving the Antioxidative Capacity and Intestinal Health in the Chinese Soft-Shelled Turtle (*Pelodiscus sinensis*)

**DOI:** 10.3390/antiox14010112

**Published:** 2025-01-20

**Authors:** Liqin Ji, Yisen Shangguan, Chen Chen, Chengqing Wei, Junxian Zhu, Xiaoyou Hong, Xiaoli Liu, Xinping Zhu, Wei Li

**Affiliations:** Key Laboratory of Tropical and Subtropical Fishery Resources Application and Cultivation, Ministry of Agriculture and Rural Affairs, Pearl River Fisheries Research Institute, Chinese Academy of Fishery Sciences, Guangzhou 510380, China; jiliqin@prfri.ac.cn (L.J.);

**Keywords:** Chinese soft-shelled turtle, tannin, antioxidant, intestinal microbiota, transcriptome, metabolome, signaling pathways

## Abstract

To investigate the effect of tannic acid (TA) on the growth, disease resistance, and intestinal health of Chinese soft-shelled turtles, individual turtles were fed with 0 g/kg (CG), 0.5 g/kg, 1 g/kg, 2 g/kg, and 4 g/kg TA diets for 98 days. Afterwards, the turtles’ disease resistance was tested using *Aeromonas hydrophila*. The results showed that 0.5–4 g/kg of dietary TA increased the growth performance and feed utilization (*p* < 0.05), with 2.38 g/kg being the optimal level for the specific growth rate (SGR). The addition of 0.5–4 g/kg of TA in diets increased the mucosal fold height and submucosa thickness of the small intestine, which reached a maximum of 2 g/kg. The addition of 0.5–2 g/kg of TA effectively reduced the cumulative mortality that had been induced by *A. hydrophila*, with the 2 g/kg dosage leading to the lowest mortality. Additionally, 1–4 g/kg of TA improved the T-SOD, CAT, and GSH-Px activities during infection, while 2 g/kg of dietary TA enhanced the richness and diversity of the microbiota, for example, by increasing Actinobacteria but inhibiting Firmicutes. The transcriptome demonstrated that the predominant differentially expressed genes (DEGs) in TA2 were mainly enriched in the PPAR signaling pathway (*Acsl5*, *Apoa2*, *Apoa5*, *Fabp1*, *Fabp2*, and *Fabp6*); in glycine, serine, and threonine metabolism (*Chdh*, *Gatm*, and *Shmt1*); and in steroid biosynthesis (*Cel*, *Hsd17b7*, *Soat2*, and *Sqle*). The main differentially expressed metabolites (DEMs) that were discovered by means of metabolome analysis included cholylhistidine, calcipotriol, 13-O-tetradecanoylphorbol 12-acetate, and hexahomomethionine in CG vs. TA2. Integrative analyses of two omics revealed that 2 g/kg of TA mitigated inflammation by activating the PPAR signaling pathway and regulating the lipid metabolism via multiple pathways, such as steroid biosynthesis and α-linolenic acid metabolism. In general, the inclusion of 2 g/kg of TA in turtle diets can optimally promote growth and bacterial resistance by maintaining intestinal health and improving antioxidant capacity.

## 1. Introduction

The Chinese soft-shelled turtle (*Pelodiscus sinensis*) is an aquatic animal of economic importance owing to its good nutritional attributes, such as its abundant protein, vitamin, and mineral contents and low fat content [1]. In addition, the Chinese soft-shelled turtle has been used in nutraceuticals in China owing to its medical benefits in treating diabetes, menopause, hypertension, and other chronic diseases [2].

This species is popular in the Asian market, including Japan, South Korea, and China [3]. To meet the huge market demand for this species in China, the production model is transitioning to factory farming, and in 2023, the production of the Chinese soft-shelled turtle in China rose to 497,536 tons [4]. However, the high density of factory farming can increase susceptibility to bacterial infections, such as *Aeromonas hydrophila*, *Citrobacter freundii*, and *Edwards iellatard* infections, which have resulted in mass mortality and huge economic losses [5]. Therefore, there is an urgent need to find safe and effective methods to prevent the Chinese soft-shelled turtle industry from succumbing to the threat of bacteria.

To prevent aquatic diseases, the World Health Organization (WHO) and the Food and Agriculture Organization of the United Nations (FAO) are pushing for the addition of natural immunostimulants in aquaculture [6]. Medicinal plants, as the typical examples of natural immunostimulants, have been shown to have beneficial effects on fish health owing to their abundant bioactive metabolites (phytochemicals) [7]. Phenolic compounds, which are considered to be some of the most effective phytochemicals, have multiple beneficial effects as an additive to fish feed, such as anti-inflammatory, antioxidative, and antimicrobial activities [8]. Tannic acid (TA), a water-soluble polyphenolic compound, can be extracted from numerous plants and their fruits [9]. The chemical formula for commercial TA is C_76_H_52_O_46_, which consists of a central glucose unit and 10 gallic acid molecules attached to it and has a molecular mass of 1701.19 g/mol [10].

Owing to its unique structure, TA exhibits various biological activities, such as promoting growth and enhancing immunity, antioxidation, and antimicrobial activity (against bacteria and viruses) [11,12,13]. It has been reported to be widely used as a feed additive in the raising of pigs, cattle, and sheep [14,15,16]. For example, supplementation with TA in weaned piglets’ diets can increase productivity by mitigating oxidative stress and stimulating gut health [17]. The addition of 1000 mg/kg of dietary TA for broilers protects the intestinal barrier, mitigates inflammatory responses, and enhances the antioxidant capacity under the co-infection of *Coccidia* and *Clostridium* perfringens [11]. Similarly, dietary TA supplements modulate the metabolites and antioxidative capacity of plasma in lambs [16]. Recently, the effect of TA on aquatic species has received increasing scientific attention. For example, Zhang et al. found that dietary TAs alter the digestion and absorption of proteins in Pacific white shrimp [18]. Yang et al. suggested that supplementing the diet with 3.75 g/kg of TAs can promote the growth performance, feed utilization, and intestinal health of largemouth bass by increasing the abundance of beneficial bacteria in their intestine [19]. In general, TA has great potential as an alternative to in-feed antibiotics to resist bacterial infection in the aquatic industry [20]. However, the application of TA as a feed additive in the Chinese soft-shelled turtle has not been reported.

The transcriptome can detect a complete set of transcripts by quantifying mRNA expression [21]. It has been a valuable tool in aquaculture for discovering and quantifying molecular markers that are associated with growth and immunological and nutritional status [22]. For example, the hepatic transcriptome of largemouth bass demonstrates that dietary *Lactobscillus plantarum* can enrich the expressed genes in the peroxisome proliferator-activated receptor (PPAR) signaling pathway and citrate cycle [23]. The metabolome can identify metabolic biomarkers for monitoring the health, stress, and nutritional status of fish, which helps us understand how biological processes respond to exogenous influences [24]. Metagenomic 16SrDNA gene amplicon sequencing can be employed to characterize the composition and abundance of microorganisms. For example, high-throughput 16SrDNA sequencing shows that the intestinal microflora plays an important role in the pathogenesis of pearl gentian grouper SBMIE [25]. It also demonstrates that dietary polysaccharides modulate the intestinal microbiota and ameliorate immunity in *Luciobarbus capito* [26]. Furthermore, the application of multi-omics can provide more detailed information than a single technique. Transcriptome and gut microbiome analyses show that *Bidens pilosa* is beneficial in promoting growth and metabolism in tilapia [27]. The joint application of transcriptome and metabolome analyses reveals that astaxanthin can help *Exopalaemon carinicauda* resist alkaline stress [28]. In addition, the integration of microbiome and metabolome analyses showed that the intestinal microbe can alter the lipid metabolism of Huanghe carp under short photoperiods [29].

Generally, TA has exhibited various beneficial effects on different animals. However, academic research on using TA for Chinese soft-shelled turtles is sparse. Herein, we speculated that TA might promote the growth performance and resistance against bacteria by improving the intestinal properties of the Chinese soft-shelled turtle. To verify this hypothesis, we performed a feeding experiment and challenge test using *A. hydrophila* to explore the effects of TA on the growth performance and resistance against bacteria of Chinese soft-shelled turtles. Furthermore, plasma antioxidative indicators, intestinal histology, transcriptome, metabolome, and 16SrDNA techniques were integrated to comprehensively examine the influence of TA on the animals’ intestinal health. This research can provide a theoretical basis for the application of TA in the Chinese soft-shelled turtle industry.

## 2. Material and Methods

### 2.1. Experimental Diets

The TA products, which were extracted from Chestnut (purity = 70%), were purchased from Pureland Co., Ltd. (Guangzhou, China). Commercial pellet diets (Guangdong Nutriera Group Co., Ltd., Guangzhou, China) without TA were used as the basal diet and were used as the diet for the control group (CG). Five isoproteic (about 462 g kg^−1^ of crude protein) and isolipidic (about 76 g kg^−1^ of crude lipid) diets were formulated, with graded levels of TA supplementation (0, 0.5, 1, 2, and 4 g kg^−1^ of the dry matter of the diet). These diets were designated as the CG, TA0.5, TA1, TA2, and TA4 diet, respectively. The formulations and proximate compositions of the experimental diets are shown in Table 1.

### 2.2. Experimental Animals and Feeding Management

**Figure 1:**
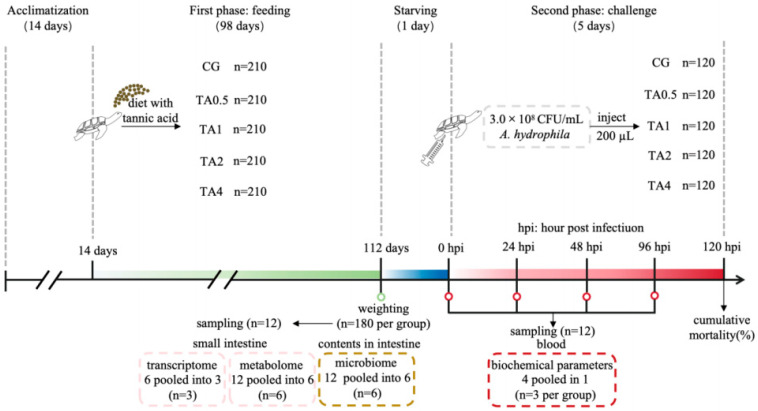
Experimental flowchart. CG indicates the control group. TA0.5 indicates the group supplemented with 0.5 g/kg TA. TA1 indicates the group supplemented with 1 g/kg TA. TA2 indicates the group supplemented with 2 g/kg TA. TA4 indicates the group supplemented with 4 g/kg TA.

The Chinese soft-shelled turtles were obtained from Huizhou Wealth Xing Industrial Co., Ltd. (Huizhou, China). The experiment consisted of two stages: the first phase was the 98-day feeding program of the turtles, followed by the second stage, which was the *A. hydrophila* infection challenge test (Figure 1). These two experiments were performed at the Pearl River Fisheries Research Institute, Chinese Academy of Fishery Sciences (Guangzhou, China). After two weeks of acclimation with commercial feed, a total of 1050 turtles with an average body weight of 12.54 ± 0.54 g and in good health were randomly selected as experimental turtles and then randomly and evenly allocated into 15 polyethylene tanks (1 m × 1 m × 0.25 m). The random selection of turtles was achieved through human choice without weight and appearance biases. In accordance with the TA dosages in the diets, five groups (CG, TA0.5, TA1, TA2, and TA4) with triplicate tanks were established.

The turtles were fed corresponding diets to apparent satiation twice a day (9:00 and 17:00) for 98 days. A small amount of pellets were left to settle in the tank, and most turtles showed no feeding behavior during the meal, which indicated an apparent satiation state. One hour after feeding, the residual bait and feces were removed. During the non-feeding period, the air pump oxygenated the water body for 6 h daily. Approximately one-third of the tank’s water was renewed every 3 days. The water temperature was maintained at 28 ± 1 °C using an electric heater, and the other water parameters were maintained as follows: pH, 8.0 ± 0.4; dissolved oxygen, 6.0 ± 1.7 mg/L; NH_3_-N, 4.0 ± 1.2 mg/L; NO_2−_, 1.0 ± 0.4 mg/L; and alkalinity, 45 ± 4.

### 2.3. Challenge Test Using A. hydrophila

The second phase was to challenge the turtles in 15 small square tanks (65 cm × 40 cm × 14.5 cm) by using *A. hydrophila,* which was originally isolated from a diseased Chinese soft-shell turtle. These bacteria were cultured in a Brain–Heart Infusion medium (Qingdao Hope Bio-technology Co., Ltd., Qingdao, China) for 24 h at 28 °C. The cultures were centrifuged at 2500× *g* for 10 min, and the supernatant was discarded. The bacteria were resuspended in sterile 0.85% NaCl, adjusting the concentration to 3.0 × 10^8^ CFU/mL under the 0.5 McFarland standard. (A 3.0 × 10^8^ CFU/mL concentration was discovered to be the median lethal concentration for turtles in a pre-experiment.) The turbidity was adjusted using a turbidimeter (Biome Rieux, Boston, MA, USA). Each individual was injected with 200 µL 3.0 × 10^8^ CFU/mL of *A. hydrophila* suspension. A total of 120 turtles in each group were infected for the challenge test. A total of 20 turtles in the CG were injected with 0.85% NaCl to ensure that this concentration did not lead to the death of turtles. The *A. hydrophila*-induced mortality was recorded every 12 h until 120 h post-infection (hpi). We then calculated the cumulative mortality as follows:

$$\mathrm{Cumulative}\;\mathrm{mortality}\;(\%)=100\%\;\times \;\frac{\mathrm{Nd}}{\mathrm{Nt}\;-\;\mathrm{Ns}}$$

where N_t_ is the initial infected turtle number and Ns is the total number of sampled turtles. N_d_ is the cumulative number of dead turtles, excluding the number of sampled turtles.

### 2.4. Sample Collection

The timeline of the sampling process is shown in Figure 1. At the end of the 98-day feeding experiment, a total of 180 individuals per group were weighed to determine their growth-related parameters. Then, 12 turtles in each group were sampled as the 0 hpi samples after 24 h of starvation. At 24, 48, and 96 hpi in the challenge test, 12 live individuals with clinical signs were sampled from each group. The turtles were anesthetized with 1000 mg/L of methane sulfonate (MS-222) solution before sampling.

Then, 800 µL blood from each turtle was collected from the neck–chest fracture section and placed into an anticoagulant tube containing 75 IU heparin sodium. To reduce individual differences, blood samples from four individuals were mixed into one tube (n = 3 per group). After preservation at 4 °C for 5 h, the blood samples were centrifuged at 4000× *g* for 20 min at 4 °C to obtain the plasma, which was stored at −80 °C for analysis of biochemical parameters. The American Veterinary Medical Foundation (AVMA) states that the application of two or more euthanasia procedures is usually recommended for reptiles. In our study, the turtles were anesthetized with 1000 mg/L of methane sulfonate (MS-222) solution and then transferred onto ice for rapid freezing before sampling. The combination of the MS-222 treatment and ice freezing euthanized the turtles effectively. After blood collection, the turtles were rapidly dissected to collect their intestinal tissue. The weight of the sampled intestine was 0.16 ± 0.05 g. The intestinal contents from 12 turtles in each group were pooled into 6 cryopreserved tubes for the detection of microbiota. According to a previous report, the intestine of the Chinese soft-shelled turtle is divided into large and small intestines [30]. A part of the small intestine (1 cm in length) from six individuals per group was fixed in Bouin’s solution for 24 h and then transferred into 75% alcohol for histological evaluation. The remaining parts of the small intestines from 12 individuals per group were pooled into 6 tubes, which were snap-frozen in liquid nitrogen and stored at −80 °C for analyses of the transcriptome and metabolome.

### 2.5. Biochemical Analysis

The activities of plasma total superoxide dismutase (T-SOD, kit number A001-1-2), catalase (CAT, A007-1-1), glutathione peroxidase (GSH-Px, A005-1-2), alkaline phosphatase (ALP, A059-2-2), and acid phosphatase (ACP, A060-2-2) were determined using the colorimetric method according to the manufacturer’s protocols for the diagnostic kits (Nanjing Jiancheng Bioengineering Institute, Nanjing, China).

### 2.6. Intestinal Histology

The small intestines (n = 6) that were stored in 75% alcohol were dehydrated using ethanol solutions at different gradient concentrations (80%, 90%, 95%, and 100%), equilibrated with xylene, and embedded in paraffin. The samples were cut into 6 μm slices with a Leica RM 2016 (Leica Biosystems Co., Ltd., Wetzlar, Germany) and were then spread onto glass slices, dewaxed in xylenes, and stained with a hematoxylin and eosin (H&E) solution. Finally, they were dehydrated and sealed. Images of the intestinal sections were observed and recorded under a Nikon eclipse Ti2 transmission electron microscope (Nikon Corporation, Tokyo, Japan). The morphological parameters, including the mucosal fold height, mucosal fold width, lamin propria, and submucosa thickness, were measured using Image J 1.44 software (National Institute of Health, Bethesda, MD, USA).

### 2.7. Intestinal Microbiome Analysis

The genomic DNA of the microbial community was extracted from the intestinal contents of the CG and TA2 groups after the 98-day feeding experiment. Each group contained six pooled replicates for the 16S rDNA analysis (n = 6). The microbial genomic DNA was extracted using the TGuide S96 Magnetic Soil/Stool DNA Kit (Tiangen Biotech Co., Ltd., Beijing, China). The hypervariable regions V3-V4 of the bacterial 16S rDNA gene were amplified with primer pairs, including 338F: 5′- ACTCCTACGGGAGGCAGCA-3′ and 806R: 5′- GGACTACHVGGGTWTCTAAT-3′. The PCR products were detected using 1.5% agarose gel and purified using an Omega DNA purification kit (Omega Inc., Norcross, GA, USA). The purified PCR products were collected and paired-end-sequenced (2 × 250 bp) on the Illumina Novaseq 6000 platform, assisted by the BioMarker (Bio-Technology Co., Ltd., Shanghai, China).

Qualified sequences sharing more than 97% similarity were categorized into one operational taxonomic unit (OTU) using USEARCH Version 10.0 [31]. Then, they were classified into different taxonomical levels (phylum, class, order, family, and genus) based on the Naive Bayes classifier, with 70% confidence. The analysis was performed on the online platform BMKCloud (https://www.biocloud.net) accessed on 10 July 2024. α diversity indexes, including Chao1, Ace, Shannon, and Simpson indexes, were used to identify the richness and diversity of the bacteria utilizing QIIME2 software. The β diversity and species complexity among the samples were analyzed by means of principal coordinate analysis (PCoA). A Student’s *t*-test was used to compare the bacterial abundance and diversity in the two groups. The linear discriminant analysis (LDA) effect size (LEfSe) was determined to search biomarkers for highly dimensional bacteria in the CG and TA2 groups.

### 2.8. Intestinal Transcriptome

#### 2.8.1. RNA Extraction, Library Preparation, and Sequencing

After the 98-day feeding experiment, the small intestines in the CG and TA2 groups were used for transcriptomic detection. The small intestines from six individuals were pooled into three mixed samples in each group (n = 3). The total RNA was extracted with TRIzol kits (Tiangen Biotech Co., Ltd., Beijing, China). The integrity and concentration of the RNA were detected, respectively, using electrophoresis in 1% agarose gels and the Nanodrop2000 (Thermo Fisher Scientific, Waltham, MA, USA).

The RNA-seq library was generated based on 2 mg of RNA and paired-end-sequenced on an Illumina NovaSeq platform (Illumina, San Diego, CA, USA) from Biomarker Technologies (Beijing, China). The clean reads were acquired by removing a series of adapter sequences and low-quality reads. Subsequently, these clean reads were aligned to the *P. sinensis* genome (https://www.ncbi.nlm.nih.gov/genome/?term=Pelodiscus+sinensis) accessed on 30 May 2024 and deposited in the Short Read Archive (SRA) of the NCBI with BioProject accession number SUB14129726.

Gene expression levels were calculated as fragments per kilobase per million mapped fragments (FPKM). The differentially expressed genes (DEGs) were defined with a fold change > 2 and adjusted *p*-value < 0.05. The DEGs were functionally annotated with the gene ontology (GO) and Kyoto Encyclopedia of Genes and Genomes (KEGG) databases.

#### 2.8.2. Validation of the Transcriptomic Results

The transcriptomic data were validated using quantitative real-time PCR (qRT-PCR). The RNA samples that were used in the qRT-PCR were identical to those in the transcriptome. A total of 10 DEGs were randomly selected to validate the RNA-seq data. All primers that were designed for the qRT-PCR are described in Table S1. The reaction system contained 2 µL of cDNA (~1 µg), 10 µL of 2 × SYBR Green Master Mix (Takara, Dalian, China), 4 µM of each primer, 0.4 µL of ROX reference dye, and RNase-free water to a final volume of 20 µL. The qRT-PCR program was run in the ABI StepOnePlus System (Applied Biosystems, Foster, Schaumburg, IL, USA) as follows: 95 °C for 10 min, followed by 40 cycles of 95 °C for 5 s, and 60 °C for 30 s. The gene expression level was normalized with the *β-actin* gene. The fold changes in mRNA levels in the treatment group relative to the control group were calculated using the 2^−ΔΔCT^ method [32].

### 2.9. Metabolomic Analysis

#### 2.9.1. Metabolic Extraction and LC-MS Analysis

After the 98-day feeding experiment, the six pooled small intestines in each of the CG and TA2 groups were extracted for the metabolomic analysis (n = 6). The metabolites were extracted from small intestinal samples of about 50 mg with a mixed solution (methanol/acetonitrile/water = 2:2:1) containing an internal standard (L-2-chlorophenylalanine, 1 ppm); they were then vortexed for 30 s, homogenized at 45 Hz for 10 min, ultrasonicated for 10 min, and incubated on ice for 15 min. After being stored at −20 °C for 1 h, the samples were centrifuged at 12,000× *g* for 15 min at 4 °C. A total of 60 μL of supernatants was utilized for the analysis of the liquid chromatography–tandem mass spectrometry (LC-MS) system. The LC-MS system contained the ultra-high-performance liquid chromatography (UHPLC) system Waters Acquity I-Class PLUS (Waters Corporation, Milford, MA, USA), which was connected with the high-resolution mass spectrometer Waters Xevo G2-XS QT (Waters Corporation, MA, USA). After separation of the different metabolites by means of UHPLC, the primary and secondary mass spectrometry data were acquired using the mass spectrometer, which was regulated using the MassLynx V4.2 acquisition software (Waters Corporation, MA, USA). Each sample was processed using electrospray ionization (ESI) using the previously described parameters [33].

#### 2.9.2. Data Processing and Multivariate Statistical Analysis

The processing of the raw data included peak extraction, peak alignment, and metabolic quantitation using the Progenesis QI software (Waters Corporation, MA, USA) accessed on 1 June 2024. The metabolites were identified using public databases, namely HMDB (http://www.hmdb.ca/), Metlin (http://metlin.scripps.edu/index.php), Massbank (http://www.massbank.jp/), LIPID MAPS (https://lipidmaps.org/), mzCLOUD (https://www.mzcloud.org/), and BioNovoGene database (http://www.bionovogene.com), which were all accessed on 15 June 2024. The multivariate statistical analyses, including partial least squares discriminant analysis (PLS-DA) and orthogonal partial least squares discriminant analysis (OPLS-DA), were carried out using the R language package. The permutation tests and cross-validation were performed to separately verify the accuracy and reliability of the two models. The DEMs in the CG vs. TA2 comparison were filtered based on the criteria of a fold change > 2, projections of important variables (VIPs) > 1, and *p* < 0.05. The DEMs were functionally annotated using the KEGG databases.

### 2.10. Joint Analysis of Transcriptome and Metabolome

The DEGs (FDR < 0.05 and |log2FC| > 1) and DEMs (VIP > 1 and *p* < 0.05) were integrated for the pairwise comparisons. A Pearson model was established to evaluate the association between the DEGs and DEMs, which was assessed using the Pearson correlation coefficient (PCC) and the relevant *p*-value. Those with |PCC| > 0.80 and *p* < 0.05 were considered significantly correlated. Furthermore, the DEGs and DEMs were mapped to the KEGG database to identify their common pathways. Finally, the network of the DEGs and DEMs was described to reveal the potential signaling pathways that are regulated by TAs.

### 2.11. Statistical Analysis

All statistical analyses were performed with the GraphPad Prism 10 software (GraphPad Software Inc., La Jolla, CA, USA). To identify appropriate statistical methods, the normality of distribution was assessed using the Shapiro–Wilk test, and the homogeneity of variances was assessed using Levene’s test for all data. A one-way analysis of variance (ANOVA), followed by Duncan’s post-hoc test, was performed to detect the difference in the intestinal histological parameters. A two-way ANOVA, followed by Duncan’s post-hoc test, was performed to analyze the cumulative mortality and plasma biochemical parameters. The qRT-PCR validation data and four α diversity indexes in the intestine microbiome were analyzed using Student’s *t*-test. An unpaired *t*-test was conducted for the qRT-PCR validation data, while a Mann–Whitney U test (nonparametric method) was conducted for the four α diversity indexes. Values are presented as mean ± standard error (SE). Statistical significance was set at *p* < 0.05.

### 2.12. Ethics Statement

All experiments were performed following the Guidelines for the Care and Use of Laboratory Animals in China and were approved by the Ethics Committee of the Pearl River Fisheries Research Institute, Chinese Academy of Fishery Sciences (approval number: LAEC-PRFRI-2023-06-27; approval date: 27 June 2023). The euthanasia of the turtles was guided by the methods of AVMA, which combined MS-222 treatment and ice freezing to effectively place the turtles in an anesthetic state.

## 3. Results

### 3.1. Growth Performance and Survival Rate

**Figure 2:**
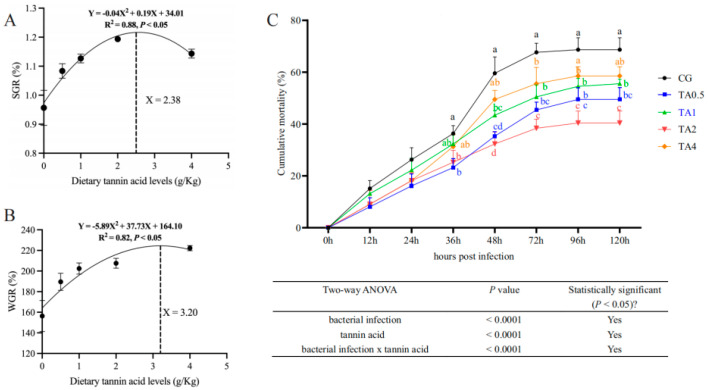
Quadratic regression analysis of the SGR (**A**) and WGR (**B**) of Chinese soft-shelled turtles who were fed a diet with graded levels of TA for 98 days. (**C**) The cumulative mortality of Chinese soft-shelled turtles who were infected with *Aeromonas hydrophila*. Different letters indicate significant differences among groups at the same point (*p* < 0.05). CG indicates the control group. TA0.5 indicates the group supplemented with 0.5 g/kg TA. TA1 indicates the group supplemented with 1 g/kg TA. TA2 indicates the group supplemented with 2 g/kg TA. TA4 indicates the group supplemented with 4 g/kg TA.

The growth performance of the juvenile turtles after the feeding trial is shown in Table 2. Compared with the CG, the final body weight (FBW), weight gain rate (WGR), specific growth rate (SGR), feed efficiency ratio (FER), and protein efficiency ratio (PER) were significantly increased in all the TA groups (*p* < 0.05; Table 2 and Table S2). Moreover, the WGR, SGR, and FER in the TA2 and TA4 groups were significantly higher than in the other groups (*p* < 0.05). In contrast, the feeding rates (FRs) in the TA2 and TA4 groups were lower than those of the other groups (*p* < 0.05). The SGR and WGR displayed a quadratic increase with the increasing dietary TA level (*p* < 0.05; Table 3), with the 2.38 g/kg and 3.20 g/kg TA levels, respectively, being predicted to have the highest values of SGR (Figure 2A) and WGR (Figure 2B).

During the challenge test (Figure 2C and Table S3), the cumulative mortalities in all groups increased gradually from 12 hpi to 72 hpi and then reached their stable highest values at 96 hpi and 120 hpi. There were no significant differences between the cumulative mortalities of the five groups from 0 hpi to 24 hpi (*p* > 0.05). The cumulative mortalities in the TA0.5, TA1, and TA2 groups were significantly lower than that in the CG from 48 hpi to 120 hpi (*p* < 0.05). Moreover, the cumulative mortality in the TA4 group declined significantly at 72 hpi in comparison with the CG (*p* < 0.05). The final cumulative mortalities of the CG, TA0.5, TA1, TA2, and TA4 groups at 120 hpi were 68.7%, 49.5%, 55.6%, 40.4%, and 58.6%, respectively, suggesting that the addition of 2 g/kg of dietary TA led to the lowest cumulative mortality.

### 3.2. Plasma Biochemical Parameters

**Figure 3:**
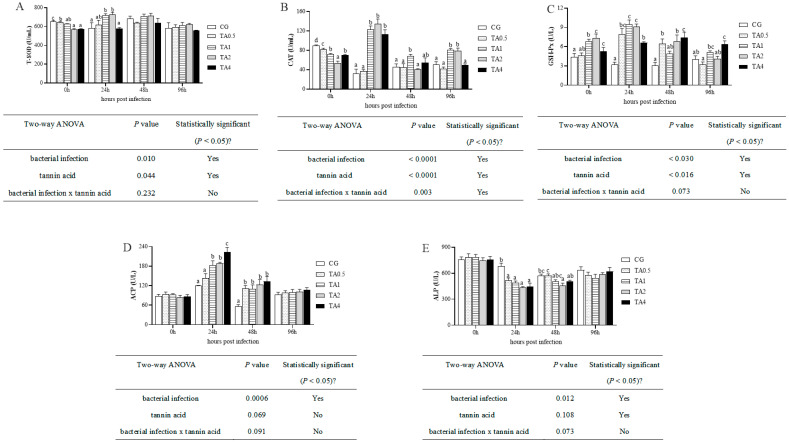
The effect of TA on plasma T-SOD (**A**), CAT (**B**), GSH-Px (**C**), ACP (**D**), and ALP (**E**) activities. All data are represented as mean ± SE (n = 3). Different letters indicate significant differences among groups at the same point (*p* < 0.05). T-SOD, total superoxide dismutase; CAT, catalase; GSH-Px, glutathione peroxidase; ACP, acid phosphatase; ALP, alkaline phosphatase. CG indicates the control group. TA0.5 indicates the group supplemented with 0.5 g/kg TA. TA1 indicates the group supplemented with 1 g/kg TA. TA2 indicates the group supplemented with 2 g/kg TA. TA4 indicates the group supplemented with 4 g/kg TA. The x-axis represents the number of hours post-infection with *Aeromonas hydrophila*.

Three plasma antioxidants (T-SOD, CAT, and GSH-Px) and two antimicrobial (ACP and ALP) enzymes were detected, as shown in Figure 3. After the 98-day feeding trial, the plasma T-SOD (Figure 3A and Table S4) and CAT (Figure 3B and Table S5) activities had decreased, while the GSH-Px (Figure 3C and Table S6) activity had increased in the TA1, TA2, and TA4 groups compared with the CG. No differences were found among the five groups for the ACP (Figure 3D and Table S7) and ALP (Figure 3E and Table S8) activities. During the challenge test, the T-SOD activities in the TA1 and TA2 groups at 24 hpi were higher than in the other groups (*p* < 0.05), whereas there was no difference among the five groups after 48 hpi (*p* > 0.05). The plasma CAT, GSH-Px, and ACP activities in the TA1 and TA2 groups rose to the highest levels at 24 hpi compared with the CG (*p* < 0.05) and dropped to lower levels after 48 hpi. In the TA4 group, the CAT activities at 24 hpi, GSH-Px activities from 24 hpi to 96 hpi, and ACP activities from 24 hpi to 48 hpi were elevated in comparison with those in the CG (*p* < 0.05). In contrast, the ALP activities in the four TA groups had significantly declined at 24 hpi compared with the CG (*p* < 0.05), but they increased to the initial levels at 96 hpi in all groups (*p* > 0.05).

### 3.3. Intestinal Morphology

**Figure 4:**
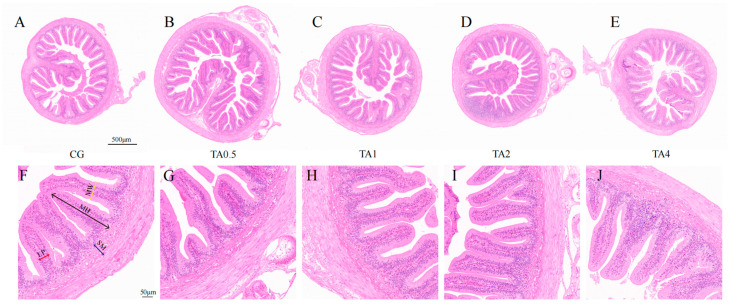
Images of the small intestinal morphology, observed using an optical microscope, in Chinese soft-shelled turtles after the 98-day feeding experiment. CG (**A**,**F**) indicates the control group. TA0.5 (**B**,**G**) indicates the group supplemented with 0.5 g/kg TA. TA1 (**C**,**H**) indicates the group supplemented with 1 g/kg TA. TA2 (**D**,**I**) indicates the group supplemented with 2 g/kg TA. TA4 (**E**,**J**) indicates the group supplemented with 4 g/kg TA. Scale bar = 500 μm (**A**–**E**). Scale bar = 50 μm (**F**–**J**). MH indicates the mucosal fold height, MW represents the mucosal fold width, LP denotes the lamin propria width, and SM denotes the submucosa.

The influence of TA on the histological parameters of the small intestine is shown in Table 4 and Figure 4. After the 98-day feeding experiment, there was no significant difference in the mucosal fold width and lamin propria among the five groups (*p* > 0.05) (Table S9). However, the mucosal fold height was significantly higher in the four TA groups than in the CG (*p* < 0.05). The mucosal fold height (868.37 ± 32.25 μm) was the largest in the TA2 group (*p* < 0.05), followed by the TA4 group (782.60 ± 29.93 μm). Similarly, the submucosa thickness in the four TA groups was remarkably increased in comparison with that in the CG (*p* < 0.05).

### 3.4. Intestinal Microbiota

**Figure 5:**
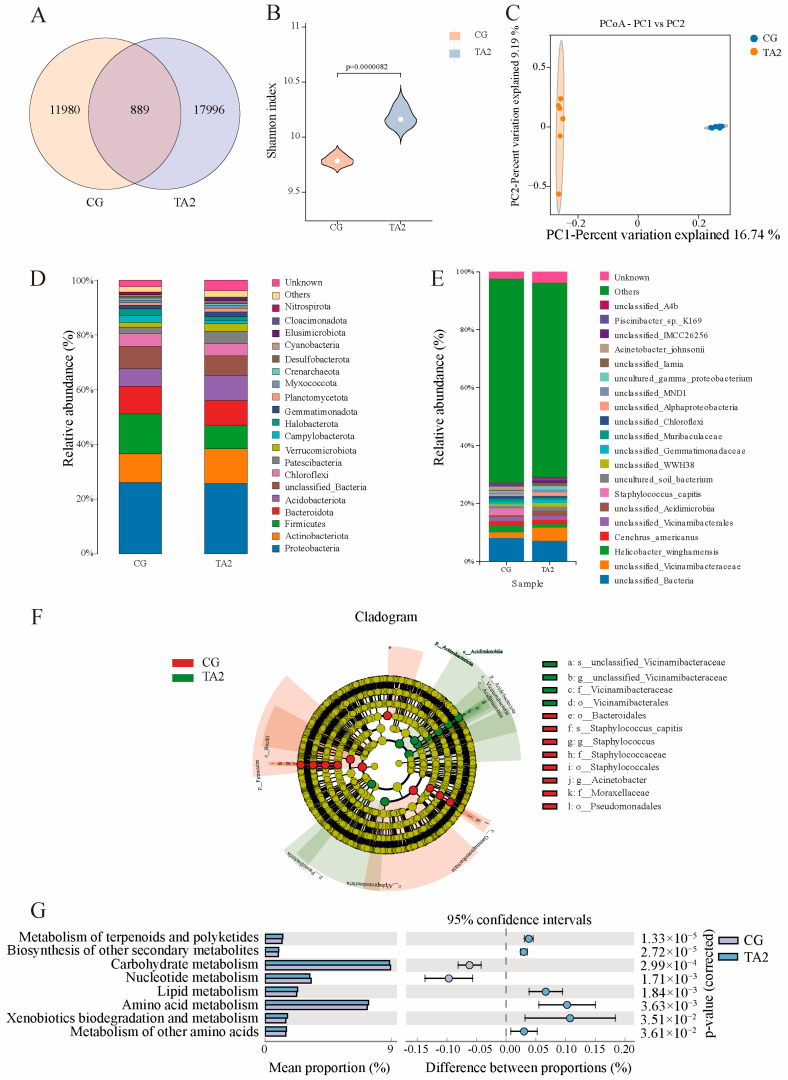
Analyses of the intestinal microbiome. (**A**) Venn diagram showing the number of OTUs that were identified in the CG and TA2 groups. (**B**) Comparison of the Shannon indexes of the CG and TA2 groups using Student’s *t*-test. (**C**) PCoA analysis of the intestinal microflora of the CG vs. the TA2 group. Intestinal microflora at the OTU (**D**) and species (**E**) levels. (**F**) LEfSe multilevel species hierarchy tree in the CG vs. the TA2 group. (**G**) A comparative KEGG pathway enrichment analysis of the microbial functional abundance in the CG and TA2 groups with PICRUSt2. *p* < 0.05 indicates a significant difference in the Shannon index. “CG” indicates the control group. “TA2” indicates the group supplemented with 2 g/kg TA.

The above results indicate that the addition of 2 g/kg of TA had better effects in terms of promoting growth performance, improving the intestinal structure, and enhancing the capacity to resist bacterial infection. To better understand the molecular mechanism of TA in the intestine function, the small intestine of the CG and TA0.2 groups were examined after the 98-day feeding trial using multi-omics analyses. The rarefaction curves of the CG and TA2 groups both reached saturation, suggesting the sufficiency of the sequencing depth for detecting most of the microbiota (Figure S1A). The rank abundance curves in the TA2 group were longer at the horizontal axis than those of the CG, indicating that the microbiota in the TA2 group had higher species richness (Figure S1B). According to the Venn diagram (Figure 5A), the CG and TA2 groups comprised 11,980 and 17,996 unique OTUs, respectively; meanwhile, they shared a total of 889 OTUs. The bacterial diversity index, including the α and β diversity of the intestine microbiota, was analyzed after OTU homogenization. No significant difference was found in the Simpson index (Figure S1C) between the two groups. However, the average values of the Shannon (Figure 5B), Chao1 (Figure S1D), and ACE (Figure S1E) indexes of the TA2 group were significantly higher than those of the CG (*p* < 0.05), reflecting the higher richness and diversity of the microbiota in the TA2 group. The PCoA model (Figure 5C) of the β diversity was established based on the OUT levels, which revealed the distinct separation of the microbial compositions of the two groups.

An analysis of the microbial communities at the phylum level (Figure 5D) showed that proteobacteria accounted for the largest percentage in the CG and TA2 groups. The Firmicutes and Actidobacteriota were the second community in the CG and TA2 groups, respectively. Additionally, *Staphylococcus*, *Helicobacter*, and *Acinetobacter* were the three largest populations in the CG, while *Vicinamibacteraceae*, *Acidimicrobiia*, and *Paucibacter* were the dominant populations in the TA2 group at the genus level (Figure 5E). An LEfSe multilevel species discriminant analysis (Figure 5F) showed that the relative richness levels of Actinobacteriota and Patescibacteria were increased, while Firmicutes was decreased at the phylum level in the TA2 group (*p* < 0.05). At the order level, Vicnamibacterales were increased; however, the Bacteroidales, Staphylococcales, and Pseudomonadales were decreased in the TA2 group compared with those in the CG (*p* < 0.05). At the genus level, the relative abundance of *Vicinamibacteraceae* was higher, but those of *Staphylococcus* and *Acinetobacter* were lower in the TA2 group compared with the CG (*p* < 0.05). At the species level, *Staphylococcus*-*capitis* was significantly lower in the TA2 group than in the CG (*p* < 0.05). To determine the functional difference that was induced by the microbial composition in the CG vs. T2 group comparison, a KEGG pathway enrichment analysis was performed using the PICRUSt2 package (Figure 5G). The level-two KEGG pathways showed that a total of eight pathways were enriched by the differential microbiota in the CG vs. the TA2 group (*p* < 0.05). These included the carbohydrate metabolism, nucleotide metabolism, lipid metabolism, amino acid metabolism, xenobiotics biodegradation and metabolism, and metabolism of other amino acids. Of these pathways, the microorganism’s abundance levels in the lipid metabolism, amino acid metabolism, and xenobiotics biodegradation and metabolism were significantly higher in the TA2 group than in the CG (*p* < 0.05).

### 3.5. Intestinal Transcriptome

**Figure 6:**
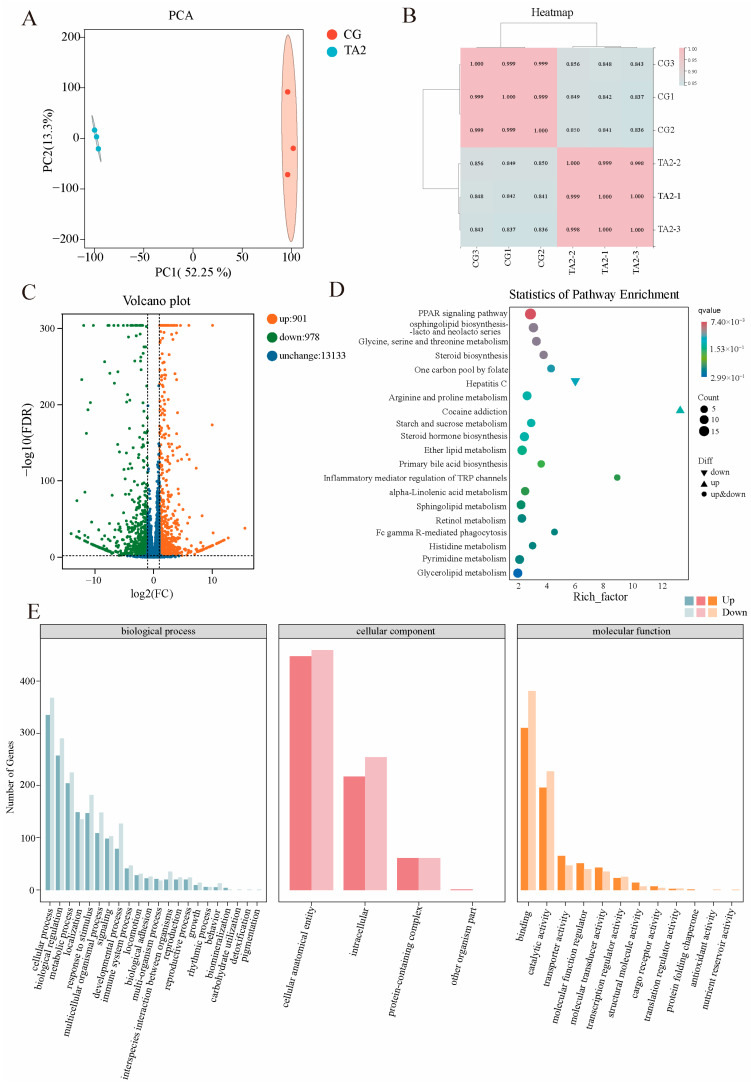
Overview of the DEGs from the transcriptome in the CG vs. TA2 group comparison. (**A**) The results of the PCA demonstrating the difference in gene expression patterns between the CG and TA2 groups. (**B**) A correlation heatmap of all the gene expression patterns in the three replicates of the CG and TA2 groups. (**C**) A volcano plot of the DEGs in the CG vs. TA2 group comparison. (**D**) A KEGG enrichment analysis of the DEGs in the CG and TA2 group comparison. (**E**) A GO enrichment analysis of the DEGs in the CG and TA2 group comparison. “CG” indicates the control group. “TA2” indicates the group supplemented with 2 g/kg TA.

A total of six RNA-seq libraries were established for the CG and TA2 groups (n = 3). A total of 69,824,740 and 67,077,939 clean reads were obtained, respectively, in the CG and TA2 groups (Table S10). The ratios of Q30 bases in all the RNA-seq libraries were more than 91.86%; meanwhile, the average mapping ratios of clean reads to the reference genome were over 80%. These results indicated that the sequencing quality met the requirements for subsequent analysis. The principal component analysis (PCA) (Figure 6A) showed that the CG and TA2 groups were clearly separated by the first principal component (PC1), accounting for 52.25% of the total variation. The heatmap (Figure 6B) exhibited a high correlation among the three replicates in each group, with the PCCs being above 0.99. The volcano plot of the DEGs (Figure 6C) identified 1879 DEGs, including 901 upregulated and 978 downregulated ones, in the CG vs. TA2 group comparison. The KEGG analysis revealed that the DEGs in the CG vs. the TA2 group (Figure 6D) were remarkably enriched in the PPAR signaling pathway (*Acsl5*, *Apoa2*, *Apoa5*, *C1qtnf7*, *Fabp1*, *Fabp2*, *Fabp6*, and *Gk*); glycosphingolipid biosynthesis (*B3galnt1*); glycine, serine, and threonine metabolism (*Chdh*, *Gatm*, *Sdsl*, and *Shmt1*); and steroid biosynthesis (*Cel*, *Dhcr24*, *Ebpl*, *Hsd17b7*, *Soat2*, and *Sqle*). The GO enrichment analysis of the DEGs (Figure 6E) found that the cellular process (GO:0009987), biological regulation (GO:0065007), and metabolic process (GO:0008152) were mostly enriched in the biological processes. In addition, the cellular anatomical entity (GO: 0110165) and intracellular entity (GO: 0005622) were the top subcategories in the cellular component, while binding (GO:0005488) and catalytic activity (GO:0003824) were the most dominant terms in the molecular function.

To determine the dependability of the transcriptomic data, 10 genes were randomly selected to be validated in the RT-PCR experiment (Table S1, Figure S2). As shown in Figure S2A, the mRNA expressions of six genes were upregulated in both the transcriptome analysis and RT-PCR, namely, *Mapk4*, *Gcg*, *Hspa5*, *Hsph1*, *Hspa8*, and *Hspa2*. The other four gene expressions—*Cel*, *Klk8*, *Wnt16*, and *Dusp4*—were downregulated in both the transcriptome analysis and RT-PCR. All gene expression trends were consistent across both methods; however, the *Mapk4*, Gcg, *Klk8*, and *Dusp4* expression levels in the RNA-seq were significantly different from those in the RT-PCR (*p* < 0.05). To further calculate the correlation of the gene expression levels based on the two detective methods, the PCC between the RT-PCR and transcriptomic results was calculated (Figure S2B). The R^2^ values of the PCC were 0.97, indicating that the mRNA levels of the RT-PCR were correlated with the transcriptomic results. This confirmed the reliability and accuracy of the transcriptome analysis.

### 3.6. Intestinal Metabolome

**Figure 7:**
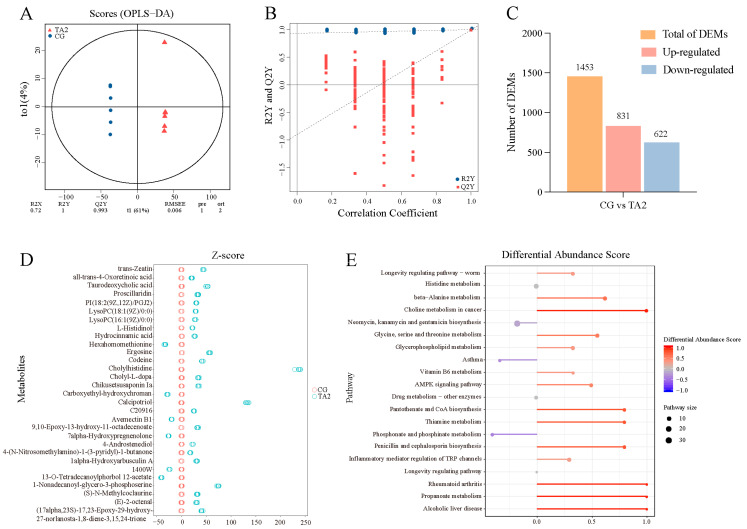
Identification of the DEMs and signaling pathways from the metabolome in the CG vs. TA2 group comparison. The cross-validation (**A**) and permutation test (**B**) of the metabolite profiles were carried out using orthogonal projection to latent structures–discriminant analysis (OPLS-DA). (**C**) The number of DEMs, filtered based on |log2 (FoldChange)| > 1 and an adjusted *p*-value < 0.05 in the CG vs. TA2 group comparisons. (**D**) Z-score plots exhibiting the top-30 DEMs from the CG vs. TA2 comparison. (**E**) The differential abundance score based on a pathway analysis of the metabolic changes in the CG vs. TA2 group comparison.

Multivariate statistical analyses, comprising PCA, PLS-DA, and OPLS-DA models, were established to detect the difference between the metabolic patterns of the CG and TA2 groups. The metabolic profiles of the CG and TA2 groups were significantly differentiated in the PCA (Figure S3A) and PLS-DA models (Figure S3B). Furthermore, an OPLS-DA plot was established to maximize the distinction between the two groups and verified with cross-validation (Figure 7A) and a permutation test (Figure 7B). The R2Y and Q2Y values of the cross-validation were, respectively, 1 and 0.993, reflecting the high predictability and lack of overfitting. The Y-intercept of Q2 < 0 in the permutation test indicated the reliability of the model.

A total of 1453 DEMs, including 831 upregulated and 622 downregulated, were identified in the TA groups compared with the CG (Figure 7C). Furthermore, a Z-score plot exhibiting the top DEMs in the CG vs. TA2 group comparison (Figure 7D) was created. Cholylhistidine, calcipotriol, 1-nonadecanoyl-glycero-3-phosphoserine, ergosine, and taurodeoxycholic acid were the five most elevated DEMs, while 13-O-tetradecanoylphorbol 12-acetate, hexahomomethionine, carboxyethyl-hydroxychroman, avemectin B1, and 7α-hydroxypregnenolone were significantly reduced DEMs in the TA2 group compared with the CG. The functional analysis of the DEMs using the KEGG database (Figure 7C) indicated that the DEMs were enriched in the propanoate metabolism, thiamine metabolism, choline metabolism in cancer, and pantothenate and CoA biosynthesis in the CG vs. TA2 group comparison.

### 3.7. Integrative Analysis of Transcriptome and Metabolome

**Figure 8:**
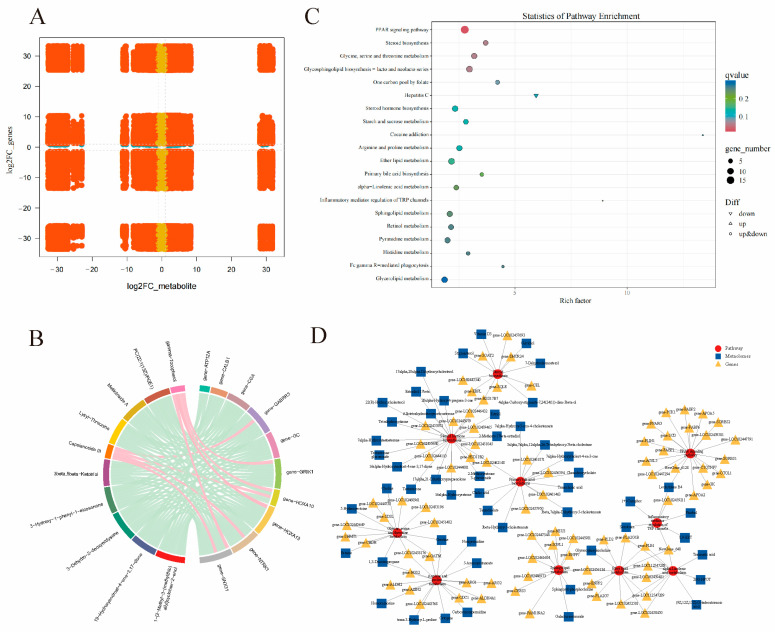
Joint analysis of the DEGs and DEMs in the CG vs. TA2 group comparison for the small intestine. (**A**) A nine-quadrant diagram indicating the correlation of the DEGs and DEMs in the CG vs. TA2 group comparison. The DEGs and DEMs with the absolute value of fold change ≧ 2 were marked with red color, which, with the absolute value of fold change < 2, were marked with yellow color. (**B**) A chord diagram exhibiting the significant association of DEGs with DEMs in the CG vs. TA2 group comparison. (**C**) Conjoint analyses of the DEG and DEM-enriched KEGG pathways. (**D**) The interactive networks of the DEGs and DEMs in the dominant KEGG pathways. “CG” indicates the control group. “TA2” indicates the group supplemented with 2 g/kg TA.

An integrated analysis of the transcriptomic and metabolomic data was performed on the BMK Cloud Platform (www.biocloud.net) accessed on 23 August 2024 to establish the correlation between DEGs and DEMs (Figure 8). The relationship of all DEGs and DEMs in the CG vs. TA2 group comparison was determined using nine-quadrant diagrams (Figure 8A). The DEMs and DEGs were negatively related in quadrants 1 and 9, while they were positively associated in quadrants 3 and 7, with |PCC| > 0.80 and *p* < 0.05. The highly correlated DEGs with DEMs are further shown in a chord diagram in Figure 8B. For example, it was found that the γ-tocopherol was positively associated with the *Grik1*, *Hoxa10*, *Hoxa13*, and *Snx31* genes; meanwhile, capsianoside III was positively related to the *Gabrr3*, *Hoxa10*, *Ntrk1*, and *Snx31* genes. In addition, the DEMs, including PC (22:1 (13Z)/PGE1), melilotoside A, lysyl-threonine, 3β, 5β-ketotriol, 3-hydroxy-1-phenyl-1-eicosanone, and 3-dehydro-2-deoxyecdysone, showed negative correlations with multiple DEGs, namely, *Atp12a*, *Calb1*, *Cga*, *Gabrr3, Gc*, *Grik1*, *Hoxa10*, *Hoxa13*, *Ntrk1*, and *Snx31*. The DEGs and DEMs were jointly enriched in the KEGG database to find their common signaling pathways (Figure 8C). The prominently enriched pathways comprised the PPAR signaling pathway; inflammatory mediator regulation of TRP channels; steroid biosynthesis; glycine, serine, and threonine metabolism; steroid hormone biosynthesis; lipid metabolism; α-linolenic acid metabolism; and primary bile acid biosynthesis. The interactive networks of the DEGs and DEMs in these pathways are shown in Figure 8D.

## 4. Discussion

### 4.1. TA Improves Growth Performance and Disease Resistance

In this study, supplementation with 0.5–4 g/kg of hydrolysable TA significantly increased the values of FBW, WGR, and SGR compared with the control group. The elevated FER and PER indexes suggest that this improved growth performance might be attributed to the increased feed utilization in the TA groups. Moreover, the effects of TA on the SGR and WGR were dose-dependent and were predicted to have the maximum effect at the 2.38 g/kg and 3.20 g/kg TA levels, respectively, rather than at the 4 g/kg level. This may be attributed to the negative effects of a high dosage of tannin on feed palatability and nutrient digestibility. Dose-dependent effects of tannins on growth performance have also been found in broilers, whose growth performance was enhanced with 0.05–0.075% TA but inhibited with 0.375% TA or higher dosages [34]. A similar study reported that the addition of 3.75 g/kg of dietary TA can improve the growth performance and feed conversion ratio in largemouth bass [19]. Meanwhile, 1.0 g/kg of dietary TA mitigates the growth retardation that is caused by oxidized fish oil and improves the feed conversion ratio in spotted seabass [35]. The intestine is an important tissue carrying out nutritional digestion and absorption [36]. The morphology of the small intestine is a crucial parameter for evaluating intestinal function [36]. The height of the mucosal fold is positively associated with the absorption efficiency of nutrients in the small intestine [37]. Herein, the mucosal fold height of the small intestine was significantly increased following the addition of 0.5–4 g/kg of dietary TA in a dose-dependent manner. Likewise, 250 mg/kg of dietary TA was shown to improve the villus height of the intestine in broilers [13]; meanwhile, the addition of 1.5 g/kg and 3.75 g/kg of TA was also shown to improve the villus height of the juvenile largemouth bass [19]. Our results indicated that dietary TA might promote feed utilization by increasing the mucosal fold height of the small intestine, which further contributed to the improved growth performance of Chinese soft-shelled turtles.

The present challenge test found that the addition of 0.5–2 g/kg of TA effectively decreased the cumulative mortality that was induced by *A. hydrophila*. In particular, the cumulative mortality in the CG (68.7%) was the largest, while that in the TA2 group was the lowest (40.4%) of the five groups. Similarly, supplementation with 0.15% hydrolysable TA can improve the survival rate of juvenile Pacific white shrimp who are infected by *Vibrio parahaemolyticus* [38]. The addition of 1000 mg/kg of dietary TA enhances the resistance capacity of broilers to co-infection with coccidia and *C. perfringens* [11]. The antimicrobial activities of TA have been verified in bacteria, fungi, and yeasts. The antimicrobial mechanisms of TA mainly consist of the inhibition of extracellular microbial enzymes, disruption of the microbial cell wall and membranes by increasing the permeability, and consumption of substrates that are essential for microbial growth [39]. In brief, the current research indicated that the addition of 2 g/kg of TA effectively enhances the resistance against *A. hydrophila* infection in Chinese soft-shelled turtles.

When organisms are challenged by external stimuli, such as pathogens and hostile environments, huge amounts of reactive oxygen species (ROS) will be produced [40]. An excess of ROS will trigger antioxidant imbalance, mitochondrial dysfunction, and lipid peroxidation [41]. The SOD, CAT, and GSH-Px, as members of the antioxidant system, can remove excessive ROS and maintain a balanced antioxidant status in cells [42]. The antimicrobial enzymes ACP and AKP play important roles in the host defense and are thus commonly used indicators for evaluating a host’s non-specific immunity [43]. In our study, the T-SOD, CAT, GSH-Px, and ACP activities were significantly increased, whereas the ACP activity was inhibited by the addition of 1–4 g/kg of TA at 24 hpi or 48 hpi. These results suggested that dietary TA can enhance the activities of antioxidative and antimicrobial enzymes, which help resist bacterial infections in Chinese soft-shelled turtles.

### 4.2. TA Affects the Intestinal Microbiota

The intestine harbors large amounts of endogenous microorganisms, which interact with the host and play an important role in the host’s growth, metabolism, and immunity [44]. It has been reported that antioxidative substances can affect a host’s health status by changing the composition of its intestinal microbiota [45]. A host’s α-diversity, which is used to assess the richness and diversity of the intestinal microbiota, can affect microbial homeostasis. In this study, the α-diversity indexes, including Ace, Chao1, and Shannon, were significantly increased in the TA2 group, suggesting that supplementation with 2 g/kg of TA can improve the richness and diversity of the microbiota in Chinese soft-shelled turtles. Similar results have been reported previously for the juvenile Pacific white shrimp, in which a low concentration (≤0.4 g/kg) of TA can elevate the bacterial diversity [38]. It was found that the addition of 200 mg/kg of dietary TA improved the microbial diversity index in Japanese sea bass [35]. However, there are some reports showing contrary results to our study. For example, it was shown that 3.75 g/kg of dietary TA can reduce the abundance and diversity of the intestinal microbiota in largemouth bass [19]. These data indicated that the effect of TA on the diversity of the intestinal microbiota might be dependent on the dosage of TA and the feeding species.

The Venn diagram created in this study showed that the number of OUTs in the TA2 group was much higher than that in the CG. Previous research has discovered that *Bacteroidetes*, *Proteobacteria*, *Firmicutes*, and *Fusobacteria* were the dominant phyla in healthy individuals of Chinese soft-shelled turtle, occupying more than 50% of the total microbial community [46]. Similarly to this research, our data also showed that *Proteobacteria*, *Firmicutes*, and *Bacteroidetes* were the abundant phyla in both groups, suggesting their potential role in the constitution of the intestinal microbiota and the maintenance of microbial homeostasis in Chinese soft-shelled turtles. In addition, our research showed that the addition of 0.2 g/kg of dietary TA led to an increase in the *Actinobacteriota* but a decrease in the *Firmicutes*. The abundance of intestinal *Firmicutes* has been found to be increased in infected Chinese soft-shelled turtles [46]. *Actinobacteria*, as Gram-positive bacteria, have been reported to play an important part in the maintenance of gut homeostasis, modulation of gut permeability, immune system, and metabolism [47]. Furthermore, we found that the addition of TA could lead to a decreased abundance of *Helicobacter* but elevate the abundances of *Vicinamibacteraceae* and *Acidimicrobiia* at the genus level. *Helicobacter*, which is usually considered to be pathogenic, is associated with peptic ulcer disease and is a risk factor for gastric cancer [48]. Therefore, our results indicated the addition of 0.2 g/kg of dietary TA might promote gut homeostasis and improve immunity by increasing the level of *Actinobacteriota* but inhibiting the levels of *Firmicutes* and *Helicobacter*.

The microbiota plays a crucial role in animal metabolism. In this study, the microbial abundance in the TA2 group was functionally enriched in the lipid metabolism and amino acid metabolism compared with the CG. Likewise, the addition of 3.75 g/kg of TA has been reported to regulate lipid metabolism by increasing the abundance of *Bacillus* and *Lysinibacillus* in largemouth seabass [19]. It has also been found that supplementation with 1.75 g/kg of TA decreases the fatty acid and triglyceride contents in grass carp [49]. Additionally, 2 g/kg of TA has been found to reduce total cholesterol and triglyceride levels in Chinese seabass [50]. In general, our research indicated that the addition of 2 g/kg of TA in the diet is likely to regulate the lipid metabolism by altering the relative abundance of the intestinal microbiota in Chinese soft-shelled turtles.

### 4.3. Regulative Mechanism of TA in the Small Intestine

In this study, the transcriptome and metabolome were analyzed to understand the regulating pathways of TA in the intestine. PPAR is a member of the nuclear receptor superfamily, which exerts various functions in cellular differentiation, apoptosis, metabolic disease, and lipid metabolism [51]. A recent study found that the PPAR signaling pathway also has important functions in the immune response by responding to inflammatory cytokines [52]. Our results showed that multiple genes (*Acsl5*, *Apoa2*, *Apoa5*, *Fabp1*, *Fabp2*, *Fabp6*, *Gk*, *Pcl1*, *Plin1*, and *Scd*) and one metabolite (leukotriene B4) in the PPAR signaling pathway were up-regulated following the addition of 2 g/kg of dietary TA. Similarly, it has been demonstrated that TA could effectively inhibit inflammatory cytokines by activating PPAR signaling pathways, which alleviated skin inflammation in mice [53]. Furthermore, TA has been reported to exert anti-inflammatory activities in a variety of clinical diseases, including skin cancer [54], Alzheimer’s disease [55], and myocardial infarction [56]. Therefore, the present data suggested that dietary TA has great potential to mitigate inflammation by activating the intestinal PPAR signaling pathways in Chinese soft-shelled turtles.

The lipid metabolism in the intestine is vital for supplying adequate energy, in the form of lipids, to various organs in the body. Disorders in the intestinal lipid metabolism can cause a number of diseases, such as growth retinitis pigmentosa and growth arrest [57]. The current data found that supplementation with 2 g/kg of TA can regulate multiple pathways that are involved in the lipid metabolism, such as steroid biosynthesis, steroid hormone biosynthesis, lipid metabolism, α-linolenic acid metabolism, and primary bile acid biosynthesis. A similar study found that dietary condensed TA regulates the signaling pathways relating to the lipid metabolism in the intestine of juvenile largemouth bass [19]. In addition, TA also mitigates the disorder of the lipid metabolism that is caused by T-2 toxin [58]; it has also been found to inhibit lipid metabolism in prostate cancer cells [59]. The small intestine is a crucial organ, in which the organism carries out the digestion, uptake, and intracellular re-synthesis of exogenous and endogenous lipids. Considering the enhancement of growth performance and intestinal morphology in the TA2 group, it can be deduced that the addition of 2 g/kg of TA might increase the utilization of dietary lipid by improving the intestinal structure and regulating lipid metabolism pathways, thereby improving the growth performance in Chinese soft-shelled turtles.

To further verify our pathway model, the expressions of 10 DEGs (*Mapk4*, *Gcg*, *Hspa5*, *Hsph1*, *Hspa8*, *Hspa2, Cel*, *Klk8*, *Wnt16*, and *Dusp4*) that are involved in multiple biological processes were examined by means of qRT-PCR. The results showed that the expression patterns of these DEGs in the qRT-PCR were consistent with those of the transcriptome, with PCC values of more than 0.9. *Mapk4*, *Hsph1*, *Hspa8*, *Hspa2,* and *Dusp4* were enriched in the MAPK signaling pathway. A previous study found that TA could regulate the NF-kB and MAPK signaling pathways, which further exert anti-catabolic and anti-inflammatory activities in osteoarthritis [60]. It has been shown that TA can activate the MAPK-dependent pathway to inhibit AT1R gene expression in epithelial cells in the rat liver [61]. The current research indicated that the addition of 0.2 g/kg of TA might activate the MAPK signaling pathway to regulate biological functions in Chinese soft-shelled turtles. Moreover, the highly correlated DEG levels in both the transcriptome analysis and qRT-PCR indicate that the signaling pathway models that we developed from the transcriptome analysis are reliable.

## 5. Conclusions

This research demonstrated that supplementation with 0.5–4 g/kg of dietary TA increased the growth performance, feed utilization, and intestinal mucosal fold height in Chinese soft-shelled turtles, with levels of 2.38 g/kg and 3.20 g/kg being predicted to lead to the highest SGRs and WGRs, respectively. The challenge test found that 2 g/kg of TA led to the greatest decline in the cumulative mortality that was induced by *A. hydrophila*. In addition, the addition of 1–4 g/kg of TA improved the turtles’ antioxidative capacities by increasing the T-SOD, CAT, GSH-Px, and ACP activities at 24 hpi or 48 hpi. The addition of 2 g/kg of dietary TA increased *Actinobacteriota* but inhibited *Firmicutes* and *Helicobacter* in the intestine, which could maintain gut homeostasis and improve immunity. Integrative analyses of the transcriptome and metabolome revealed that 2 g/kg of TA may mitigate inflammation by activating the PPAR signaling pathways and regulating the lipid metabolism via multiple pathways, such as steroid biosynthesis, α-linolenic acid metabolism, and primary bile acid biosynthesis.

## Figures and Tables

**Table 1:** Formulation and proximate composition of experimental diets (% of dry matter).

Ingredient	CG	TA0.5	TA1	TA2	TA4
White fishmeal 1 ^a^	40.00	40.00	40.00	40.00	40.00
White fishmeal 2 ^b^	15.00	15.00	15.00	15.00	15.00
Chicken powder	5.00	5.00	5.00	5.00	5.00
Soybean meal	9.00	9.00	9.00	9.00	9.00
Patent flour	20.50	20.43	20.37	20.23	19.97
Cassava raw starch	5.00	5.00	5.00	5.00	5.00
Soybean oil	2.00	2.00	2.00	2.00	2.00
Choline chloride	1.50	1.50	1.50	1.50	1.50
NaCl	0.20	0.20	0.20	0.20	0.20
Turtle premix ^c^	0.20	0.20	0.20	0.20	0.20
L-lysine hydrochloride	1.00	1.00	1.00	1.00	1.00
DL-Methionine	0.40	0.40	0.40	0.40	0.40
Protein	0.20	0.20	0.20	0.20	0.20
TA ^d^	0.00	0.07	0.13	0.27	0.53
Composition	Content				
Crude protein	46.24	45.38	47.78	47.97	47.62
Crude lipid	7.65	7.26	7.61	7.51	7.21
Crude fiber	0.84	0.83	0.86	0.81	0.88
Ash	12.75	12.20	12.68	12.51	12.69
Calcium	3.35	3.15	3.36	3.18	3.25
Phosphorus	2.27	2.25	2.19	2.17	2.28

Notes: “a” indicates white fishmeal containing 65% of crude protein, which was produced in the United States (North Pacific Co., Ltd., Seattle, WA, USA). “b” indicates white fishmeal containing 58% of crude protein, which was produced in Russia (Mercury Co., Ltd., Saint Petersburg, Russia). “c” indicates the turtle premix (/kg) with the following ingredients: VA, 150,000 IU; VD, 3,110,000 IU; VE, 4000 mg; VK_3_, 400 mg; VB_1_, 800 mg; VB_2_, 1450 mg; VB_6_, 2500 mg; VB_12_, 3.0 mg; VC, 16,000 mg; *D*-calcium pantothenate, 1250 mg; nicotinamide, 8000 mg; folic acid, 250 mg; D-biotin, 10 mg; inositol, 6000 mg; magnesium, 6000 mg; zinc, 4300 mg; manganese, 650 mg; copper, 410 mg; iron, 5900 mg; cobalt, 100 mg; iodine, 75 mg; and selenium, 25 mg. “d” indicates 70% TA.

**Table 2:** Growth performance and feed utilization of the Chinese soft-shelled turtles fed graded levels of TA (g/kg diet) diets. Different lowercase letters indicated significant differences among groups.

Item	Dietary TA Levels				
	CG	TA0.5	TA1	TA2	TA4
IBW (g)	12.86 ± 3.09	14.68 ± 2.04	12.63 ± 0.12	11.78 ± 1.41	12.50 ± 2.01
FBW (g)	32.77 ± 0.20 ^a^	37.68 ± 0.46 ^b^	38.19 ± 0.51 ^b^	37.87 ± 0.43 ^b^	38.42 ± 0.78 ^b^
WGR (%)	156.33 ± 8.70 ^a^	189.52 ± 4.82 ^b^	202.50 ± 3.17 ^bc^	222.28 ± 1.45 ^d^	207.50 ± 2.80 ^cd^
SGR (%)	0.96 ± 0.06 ^a^	1.08 ± 0.03 ^b^	1.13 ± 0.02 ^bc^	1.19 ± 0.01 ^d^	1.14 ± 0.02 ^cd^
FR (%)	1.65 ± 0.10 ^bc^	1.64 ± 0.03 ^bc^	1.69 ± 0.03 ^c^	1.59 ± 0.01 ^b^	1.40 ± 0.01 ^a^
FER (%)	0.54 ± 0.01 ^a^	0.61 ± 0.02 ^b^	0.61 ± 0.01 ^b^	0.77 ± 0.01 ^d^	0.65 ± 0.02 ^c^
PER (%)	1.19 ± 0.01 ^a^	1.29 ± 0.04 ^b^	1.38 ± 0.03 ^c^	1.62 ± 0.02 ^d^	1.41 ± 0.03 ^c^

**Table 3:** Regression analyses for dietary TA levels (X) vs. growth performance and feed utilization.

Regression	X_max_	R^2^	*p*
Y_FBW_ = −0.92X^2^ + 4.59X + 0.97	2.50	0.67	<0.05
Y_WGR_ = −5.89X^2^ + 37.73X + 164.10	3.20	0.82	<0.05
Y_FR_ = −0.02X^2^ + 0.03X + 1.65	0.50	0.84	<0.05
Y_SGR_ = −0.04X^2^ + 0.19X + 34.01	2.38	0.88	<0.05
Y_FER_ = −0.0008X^2^ + 0.06X + 0.56	3.93	0.95	<0.05
Y_PER_ = −0.885X^2^ + 1.357X + 1.209	3.84	0.94	<0.05

FBW, initial body weight (g/turtle); FBW, final body weight (g/turtle); WGR, weight gain rate (%); SGR, specific growth rate (%/d); FR, feeding rate (%/d); FER, feed efficiency ratio (%); PER, protein efficiency ratio (%). The values are shown with mean ± SE (n = 3). Different superscripts in the same row indicate a significant difference among groups (*p* < 0.05). The X_max_ values in the second column correspond to the highest points of the regressive equation. WGR (%) = 100 × (FBW − IBW)/IBW. SGR (%/d) = 100 × [Ln (FBW) − Ln (IBW)]/days. FR (%/d) = 100 × dry feed intake/[days × (FBW + IBW)/2]. FER (%) = fresh body weight gain/dry feed intake. PER (%) = fresh body weight gain/protein intake.

**Table 4:** The effect of dietary TA on the small intestinal microstructures of Chinese soft-shelled turtles (n = 6). Different lowercase letters indicated significant differences among groups.

Group	CG	TA0.5	TA1	TA2	TA4
Mucosal fold height (μm)	517.92 ± 39.6 ^a^	684.05 ± 30.18 ^b^	749.54 ± 22.88 ^c^	868.37 ± 32.25 ^e^	782.60 ± 29.93 ^d^
Mucosal fold width (μm)	221.90 ± 11.24	220.13 ± 3.17	198.60 ± 20.91	198.18 ± 27.18	218.22 ± 14.91
Lamin propria width (μm)	135.24 ± 8.09	127.55 ± 1.59	110.31 ± 22.46	138.63 ± 16.89	108.06 ± 11.12
Submucosa (μm)	90.98 ± 1.02 ^a^	116.78 ± 5.82 ^b^	122.18 ± 3.83 ^b^	117.34 ± 8.48 ^b^	119.82 ± 1.92 ^b^

## Data Availability

All data generated and analyzed during this study are included in the published article. All raw RNA sequencing data have been submitted to the NCBI Sequence Read Archive (SRA) with the BioProject ID PRJNA1167896.

## Supplementary Materials

The following supporting information can be downloaded at https://www.mdpi.com/article/10.3390/antiox14010112/s1, Figure S1: Intestinal microbiome analyses. (A) Rarefaction curves in CG and TA2. (B) Rank abundance curves assessing sample richness and evenness in CG and TA2. Diversity index on OUT level of intestinal microbiota of CG and TA2 comparison. (C) Simpson index. (D) Chao1 index. (E) ACE index. *p* < 0.05 indicates significant difference between two groups (C–E). “CG” indicates the control group. “TA2” indicates the group supplemented with 2 g/kg TA; Figure S2: Comparison of the transcriptome and qRT-PCR results. (A) mRNA levels of 10 genes in the transcriptome and qRT-PCR analyses. (B) Pearson correlation analysis of mRNA levels between real-time PCR and RNA-seq results in CG vs. TA2 comparison. * indicates a significant difference in mRNA levels between the transcriptome and qRT-PCR. “CG” indicates the control group. “TA2” indicates the group supplemented with 2 g/kg TA; Figure S3: Multivariate statistical analysis of the DEMs. (A) PCA and (B) PLS-DA of the DEMs in the CG vs. TA2 comparison from the metabolome. “CG” indicates the control group. “TA2” indicates the group supplemented with 2 g/kg TA.
