# Supplementary figures and images for: Dietary Tannic Acid Promotes Growth Performance and Resistance Against Aeromonas hydrophila Infection by Improving the Antioxidative Capacity and Intestinal Health in the Chinese Soft-Shelled Turtle (Pelodiscus sinensis)
Source: Antioxidants (Basel). 2025 Jan 20;14(1):112. doi: 10.3390/antiox14010112 (PMC11759827; doi:10.3390/antiox14010112)

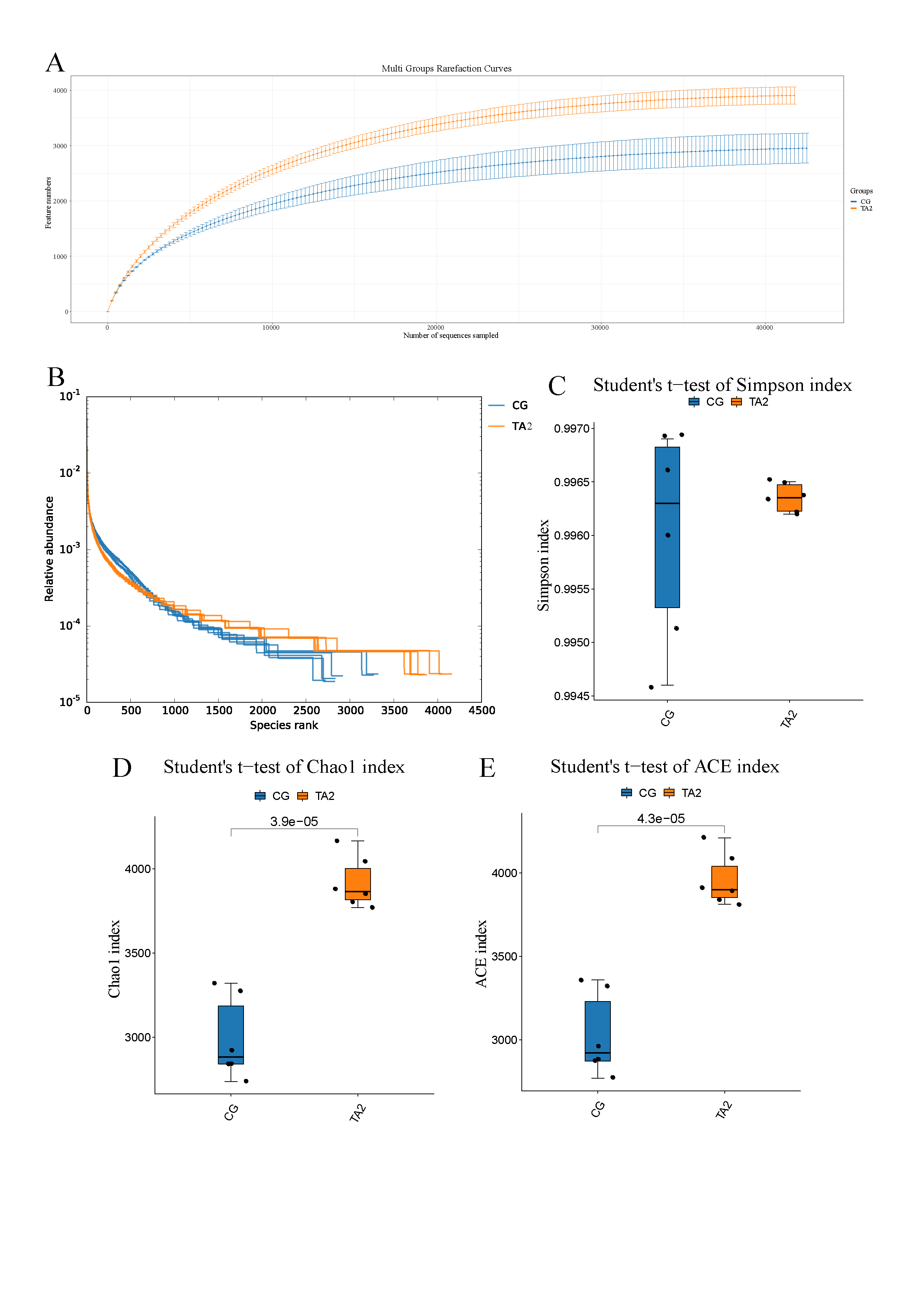

Supplement: Supplementary file 1 [file antioxidants-14-00112-s001.zip › Figure. S1.tif]

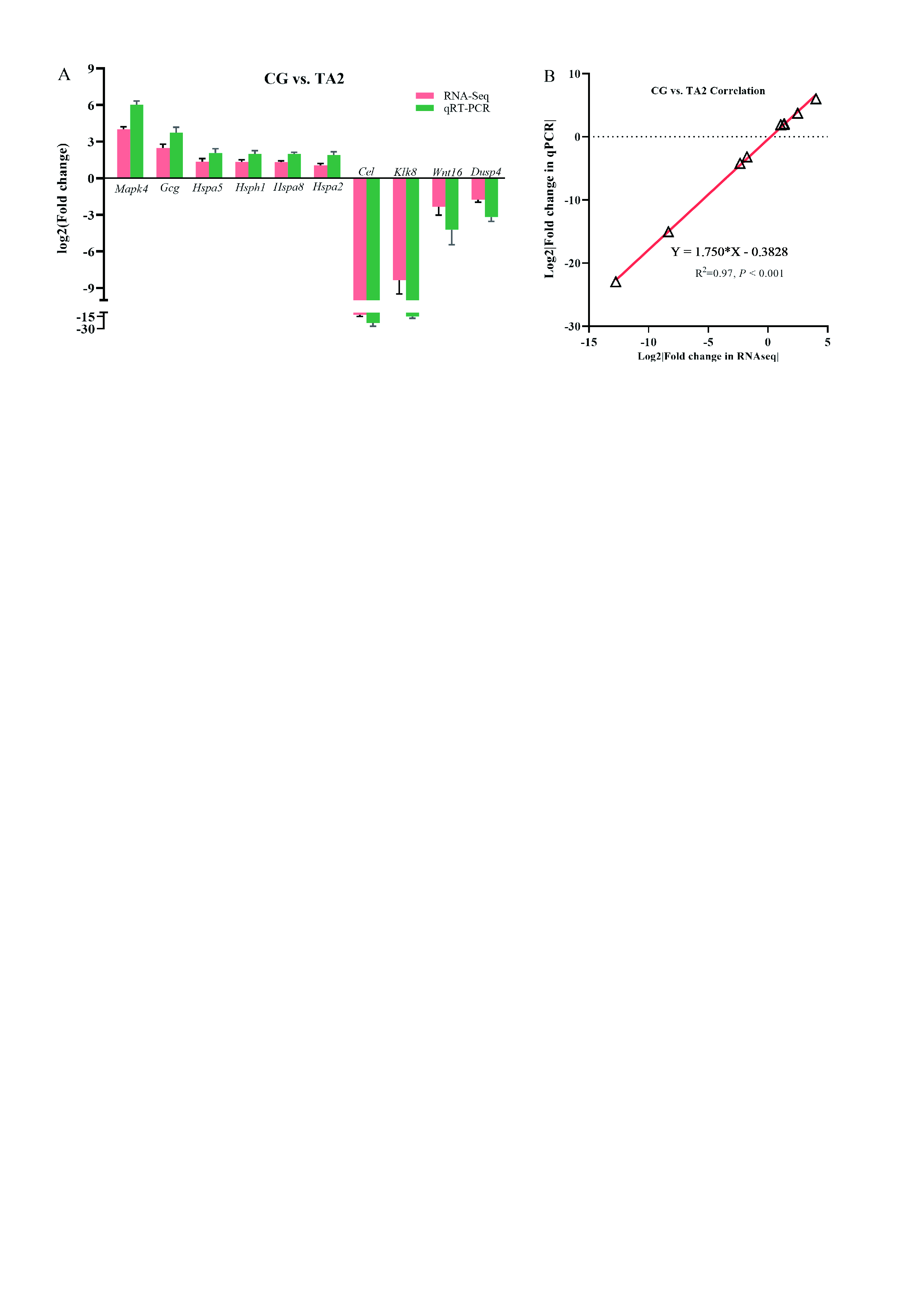

Supplement: Supplementary file 1 [file antioxidants-14-00112-s001.zip › Figure. S2.tif]

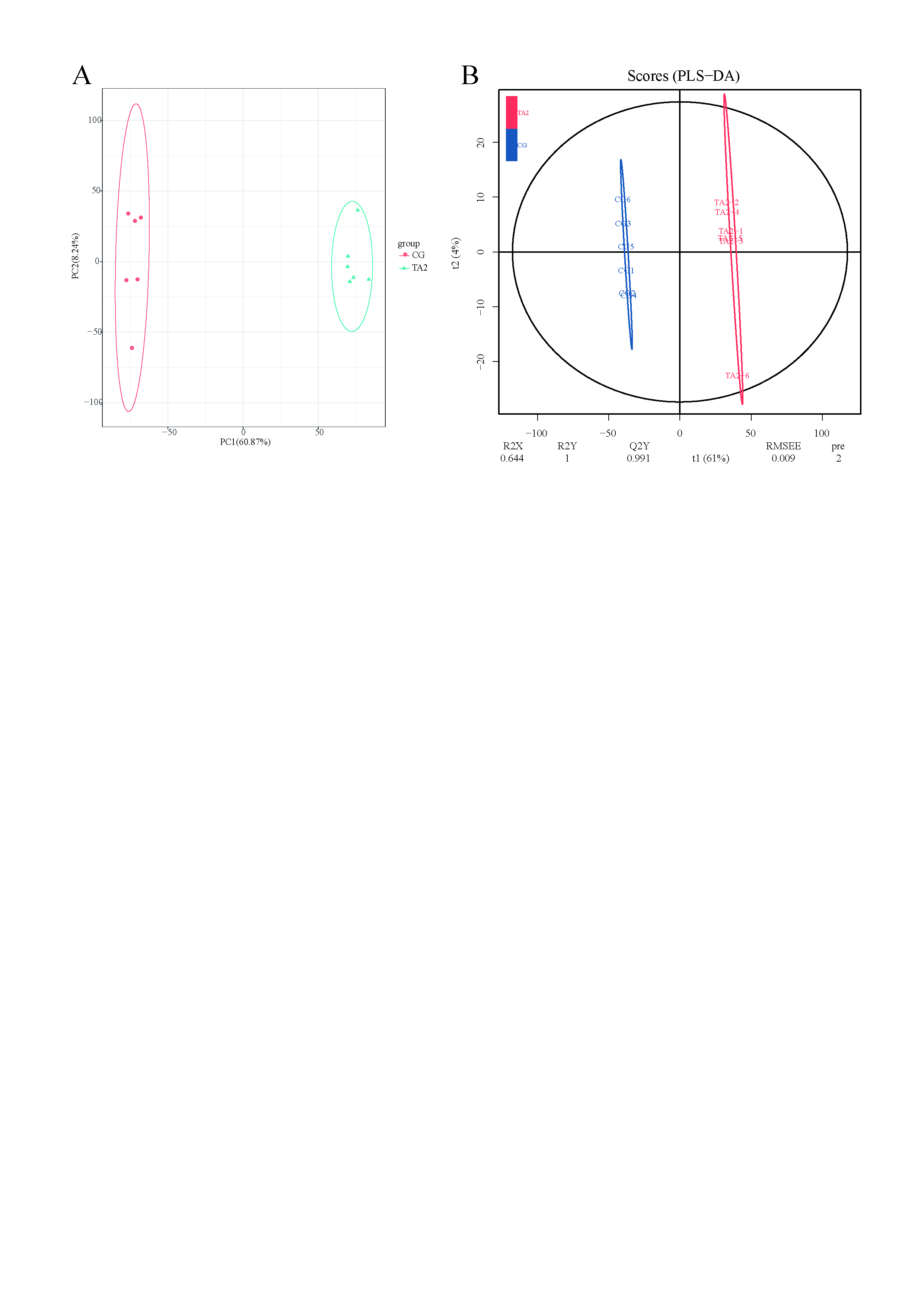

Supplement: Supplementary file 1 [file antioxidants-14-00112-s001.zip › Figure. S3.tif]
